# Space radiation measurements during the Artemis I lunar mission

**DOI:** 10.1038/s41586-024-07927-7

**Published:** 2024-09-18

**Authors:** Stuart P. George, Ramona Gaza, Daniel Matthiä, Diego Laramore, Jussi Lehti, Thomas Campbell-Ricketts, Martin Kroupa, Nicholas Stoffle, Karel Marsalek, Bartos Przybyla, Mena Abdelmelek, Joachim Aeckerlein, Amir A. Bahadori, Janet Barzilla, Matthias Dieckmann, Michael Ecord, Ricky Egeland, Timo Eronen, Dan Fry, Bailey H. Jones, Christine E. Hellweg, Jordan Houri, Robert Hirsh, Mika Hirvonen, Scott Hovland, Hesham Hussein, A. Steve Johnson, Moritz Kasemann, Kerry Lee, Martin Leitgab, Catherine McLeod, Oren Milstein, Lawrence Pinsky, Phillip Quinn, Esa Riihonen, Markus Rohde, Sergiy Rozhdestvenskyy, Jouni Saari, Aaron Schram, Ulrich Straube, Daniel Turecek, Pasi Virtanen, Gideon Waterman, Scott Wheeler, Kathryn Whitman, Michael Wirtz, Madelyn Vandewalle, Cary Zeitlin, Edward Semones, Thomas Berger

**Affiliations:** 1 Space Radiation Analysis Group, Johnson Space Center, Houston, TX USA; 2 National Aeronautics and Space Administration (NASA), Houston, TX USA; 3 Space Exploration and Mission Operations, Leidos, Houston, TX USA; 4 Institute of Aerospace Medicine, Radiation Biology Department, German Aerospace Center (DLR), Cologne, Germany; 5 Aboa Space Research Oy (ASRO), Turku, Finland; 6 KBR, Houston, TX USA; 7 European Space Research and Technology Centre (ESTEC), European Space Agency (ESA), Noordwijk, The Netherlands; 8 StemRad Inc., Tampa, FL USA; 9 Lockheed Martin Space, Houston, TX USA; 10 StemRad Ltd., Tel Aviv, Israel; 11 Department of Physics and Astronomy, University of Houston, Houston, TX USA; 12 CACI, Houston, TX USA; 13 European Astronaut Centre (EAC), European Space Agency (ESA), Cologne, Germany; 14 Present Address: Space Science and Applications (ISR-1), Los Alamos National Laboratory, Los Alamos, NM USA; 15 Present Address: Axiom Space, Houston, TX USA; 16 Present Address: Alan Levin Department of Mechanical and Nuclear Engineering, Kansas State University, Manhattan, KS USA; 17 Present Address: Oceaneering Space Systems, Houston, TX USA; 18 Present Address: The Aerospace Corporation, Houston, TX USA; 19 Present Address: Abbott Laboratories, Dallas, TX USA; 20 Present Address: Advanced Medical Physics, Inc., Houston, TX USA

**Keywords:** Applied physics, Space physics

## Abstract

Space radiation is a notable hazard for long-duration human spaceflight^1^. Associated risks include cancer, cataracts, degenerative diseases^2^ and tissue reactions from large, acute exposures^3^. Space radiation originates from diverse sources, including galactic cosmic rays^4^, trapped-particle (Van Allen) belts^5^ and solar-particle events^6^. Previous radiation data are from the International Space Station and the Space Shuttle in low-Earth orbit protected by heavy shielding and Earth’s magnetic field^7,8^ and lightly shielded interplanetary robotic probes such as Mars Science Laboratory and Lunar Reconnaissance Orbiter^9,10^. Limited data from the Apollo missions^11–13^ and ground measurements with substantial caveats are also available^14^. Here we report radiation measurements from the heavily shielded Orion spacecraft on the uncrewed Artemis I lunar mission. At differing shielding locations inside the vehicle, a fourfold difference in dose rates was observed during proton-belt passes that are similar to large, reference solar-particle events. Interplanetary cosmic-ray dose equivalent rates in Orion were as much as 60% lower than previous observations^9^. Furthermore, a change in orientation of the spacecraft during the proton-belt transit resulted in a reduction of radiation dose rates of around 50%. These measurements validate the Orion for future crewed exploration and inform future human spaceflight mission design.

## Abstract (web summary)

Measurements from the heavily shielded Orion spacecraft during the uncrewed Artemis I mission show dose-rate reductions due to shielding and orientation for Van Allen belt crossings and quantify the interplanetary cosmic-ray radiation in a human-rated spacecraft.

## Main

**Fig. 1:**
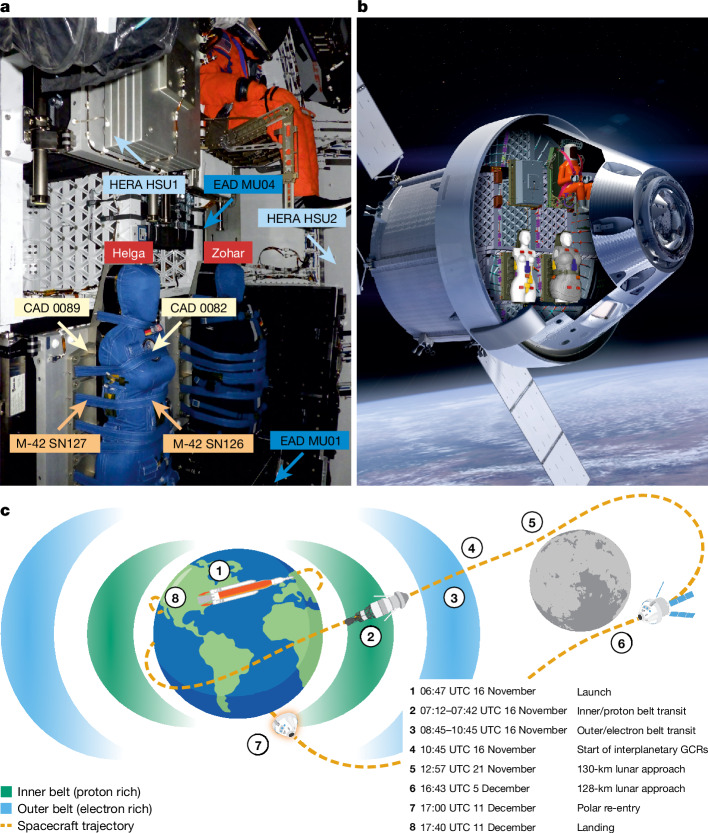
Artemis I instruments and radiation environments. **a**, Radiation instrumentation and phantoms inside Orion. These consist of the NASA HERA system, the ESA EAD system, as well as the NASA CAD and DLR M-42 instruments. The HERA system and the EADs were hard-mounted at various distinctly shielded locations in Orion. CAD and M-42 were placed on the front and back surfaces (skin) and inside (organs) (M-42) of the MARE phantoms (Extended Data Figs. 1–4). **b**, Placement of the instrumentation and hardware inside the Orion spacecraft. **c**, The Orion flight profile with respect to radiation for the NASA Artemis I mission. After launch at 06:47 UTC on 16 November 2022, Orion passed the inner (proton-dominated) and outer (electron-dominated) Earth radiation belts. Orion then ventured into interplanetary space dominated by GCRs. It passed the Moon twice on 21 November (first lunar fly-by at a distance of 130 km) and on 5 December (second lunar fly-by at a distance of 128 km). During these fly-bys, the Moon acts as a shield against GCRs. Orion re-entered Earth’s atmosphere over the South Pole and landed in the Pacific Ocean close to San Diego, California on 11 December 2022 at 17:40 UTC.

**Extended Data Fig. 1:**
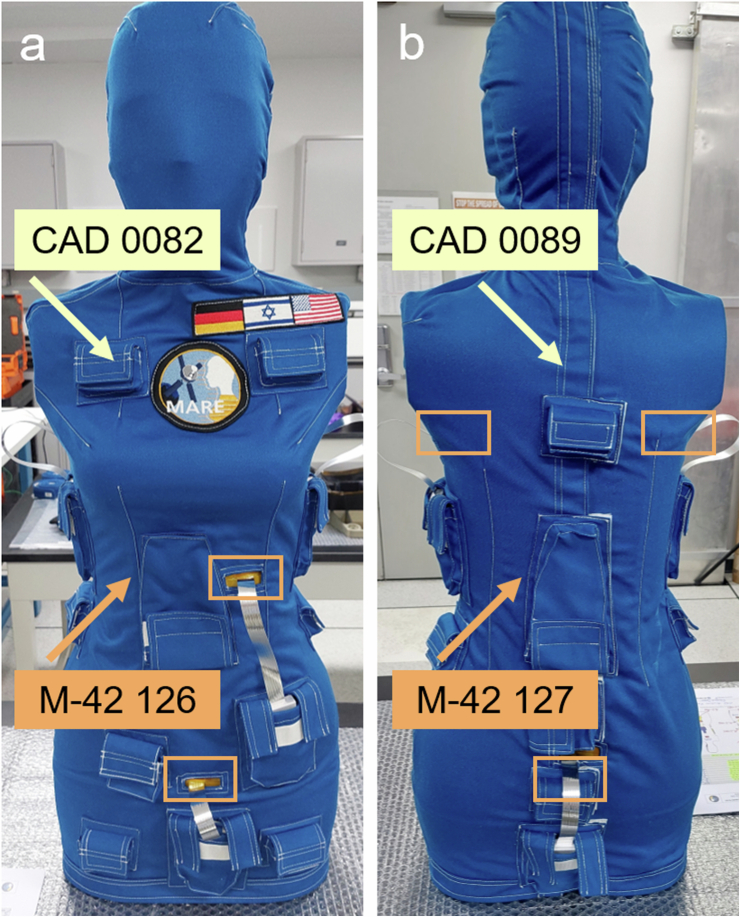
The MARE Helga phantom with mounted radiation detectors. The phantom front side (**a**) and back side (**b**) are covered with a blue poncho made of flame-retardant Nomex material, which includes pockets for the placement of the radiation detectors. CAD 0082 and M-42 SN126 were placed on the front side (skin) of the Helga phantom (lesser shielding) and CAD 0089 and M-42 SN127 were placed at the back side (skin) of the radiation phantom (higher shielding). Also, five M-42 instruments were placed with the detector head inside the most radiation-sensitive organs of the phantom (orange rectangles), namely, the left (SN144) and right (SN145) lungs, stomach (SN146), uterus (SN147) and spine (SN148). The relevant detector heads of the M-42 instruments inside the phantom are connected with a silver cable to the readout electronics, which are positioned inside the blue poncho pockets.

**Extended Data Fig. 2:**
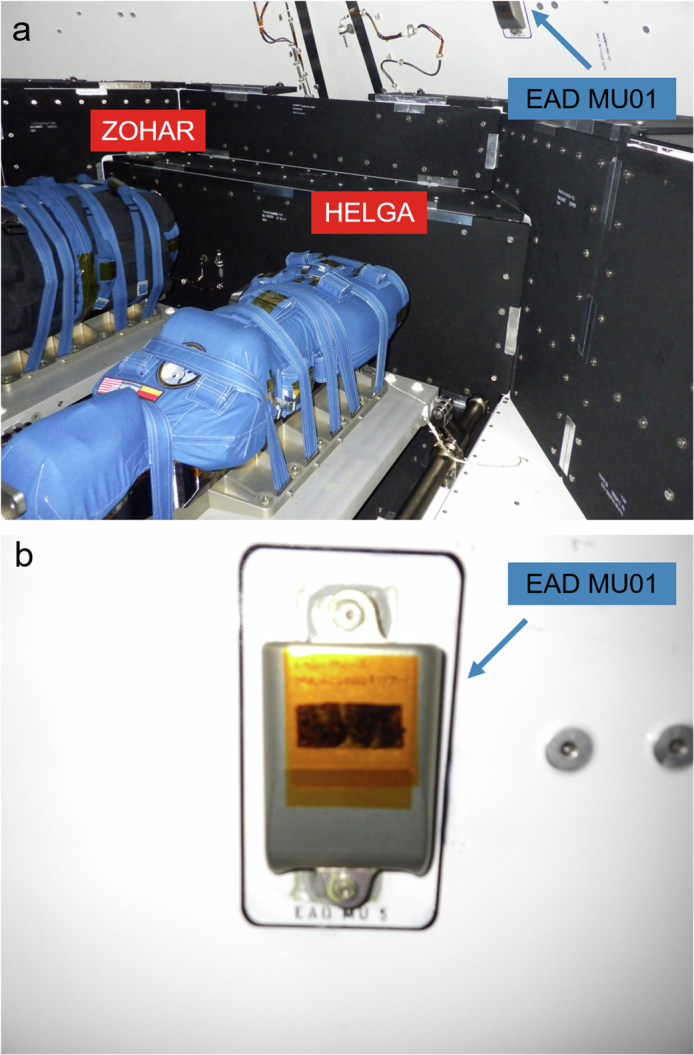
EAD MU01 placement in Orion. **a**, EAD MU01 was hard-mounted on the outer wall of Orion and is shown in relation to the MARE radiation phantoms Helga and Zohar. **b**, EAD MU01 (zoomed-in view) as hard-mounted at a low-shielding location on a blank wall in sector D inside Orion.

**Extended Data Fig. 3:**
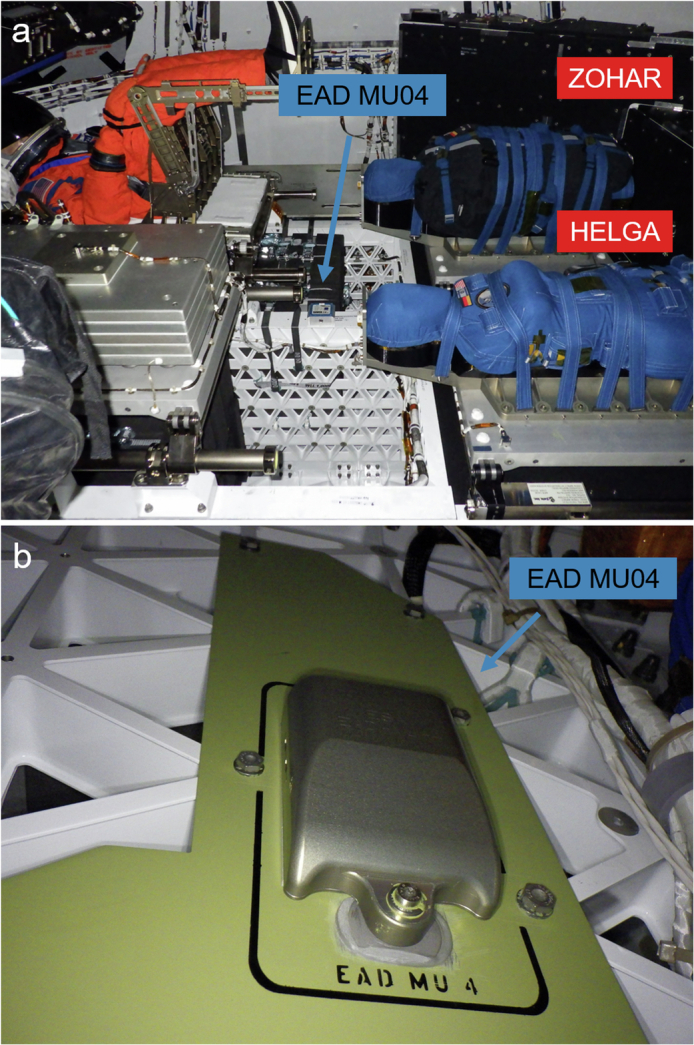
EAD MU04 placement in Orion. **a**, EAD MU04 was hard-mounted close to the HERA HPU and shown in relation to the MARE radiation phantoms Helga and Zohar. **b**, EAD MU04 (zoomed-in view) as hard-mounted at a high-shielding location below the seats in the storm shelter of Orion.

**Extended Data Fig. 4:**
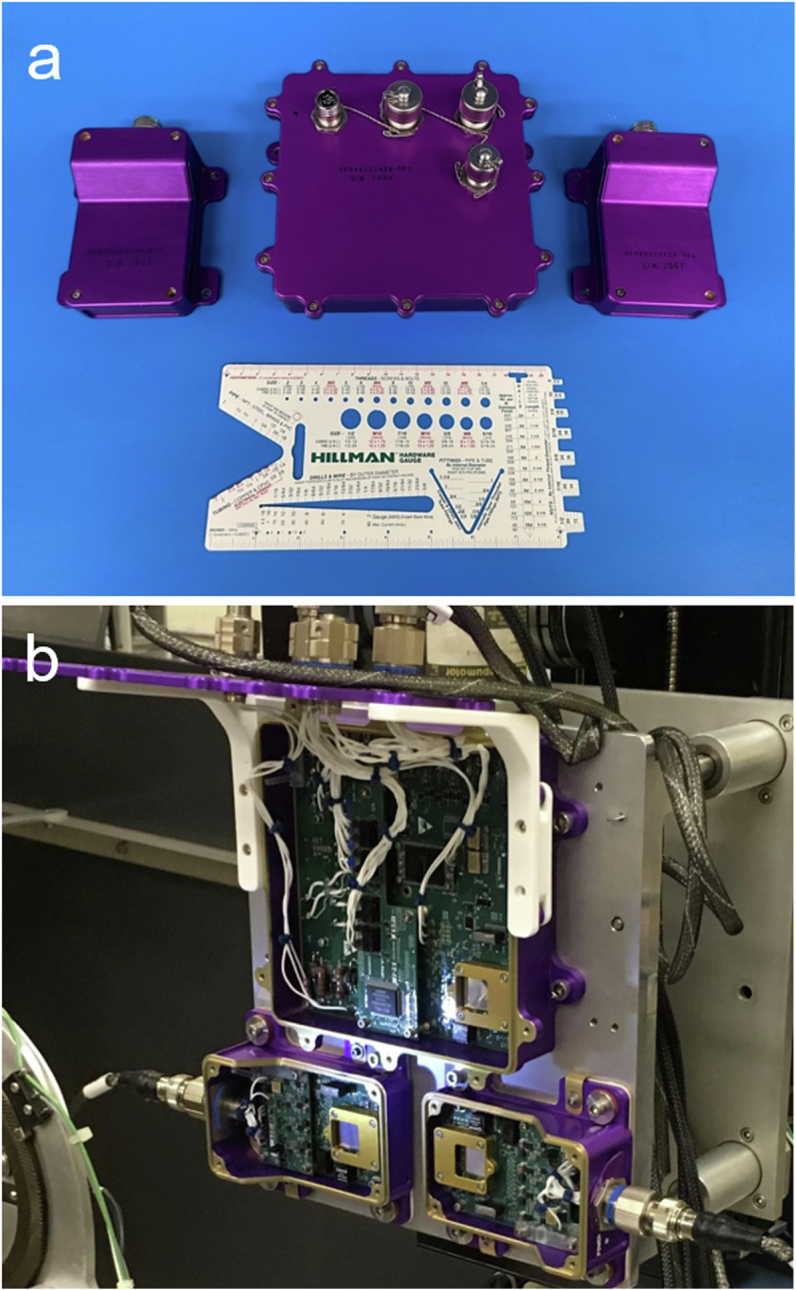
The NASA HERA system. **a**, HERA system with ruler for reference. The large box is the HPU, the smaller boxes are the two HSUs. **b**, HERA system with ‘lids open’ during calibration. The small gold boxes contain the Timepix detectors, visible as the mirror-like surfaces. HERA HSU1 is mounted in the storm shelter (high-shielded location) of Orion, HSU2 is in the crew cabin (lower shielded location). The HPU is contained inside the life support avionics box and is the most shielded HERA sensor.

Characterization of the space radiation environment in the crew cabin was a key objective of Artemis I. Radiation was assessed using detectors at fixed locations in Orion (Fig. 1a,b and Extended Data Figs. 1–4) throughout the Artemis I mission (Fig. 1c) and measured in the Matroshka AstroRad Radiation Experiment (MARE^15,16^) using instrumented life-size female radiation phantoms (Helga and Zohar) that replicate the radiation-transport properties of the human body. Here we used the NASA Hybrid Electronic Radiation Assessor (HERA)^17^, the European Space Agency (ESA) Active Dosimeter (EAD)^18^, the German Aerospace Center (DLR) M-42 (ref. ^19^) and the NASA CAD Crew Active Dosimeter (CAD)^20^ instruments. The principal experimental difference between these detectors is that HERA and EAD can be used to estimate, as well as the ionizing energy imparted or ‘absorbed dose’ in gray (Gy) measured by M-42 and CAD, the biological detriment of radiation or ‘dose equivalent’ in sievert (Sv). Human organs show markedly different radiosensitivities^21^ and, for this reason, organ doses are measured by MARE.

**Fig. 2:**
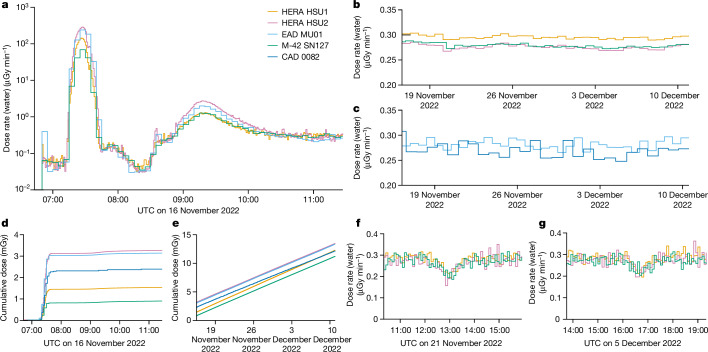
Absorbed dose measurements on Artemis I. **a**, Inner (proton-dominated) and outer (electron-dominated) belt passes as measured with the HERA, EAD and M-42 instruments. Differences in dose rate during the passes are attributed to the local shielding environments in which the detectors are placed. MU01 and HSU2 being mounted on the Orion wall have the lowest shielding. HSU1 mounted in the Orion ‘storm shelter’ has higher shielding and SN127 located at the back of the Helga phantom has the highest shielding, owing to its placement under the phantom. **b**,**c**, GCR dose rates for HERA and M-42 (**b**) and EAD and CAD for the interplanetary part of the mission (**c**). **d**, Cumulative doses for the belt passes for HERA, EAD, M-42 and CAD dominated by the proton-belt crossings (07:12–07:42 UTC), with only small contributions from the electron-belt crossing (08:45–10:45 UTC). **e**, Cumulative whole-mission dose values for HERA, EAD, M-42 and CAD reaching up to 13.47 mGy for HSU2 (Extended Data Table 1). **f**,**g**, First (**f**) and second (**g**) lunar fly-bys with the reduction in GCR dose rate owing to the shielding effect of the Moon. Source Data

Measurements of the inner proton belt show an up to fourfold difference in dose rates between the most (M-42 SN127: 69 µGy min^−1^) and the two least shielded locations (EAD MU01: 240 µGy min^−1^ and HERA HSU2: 287 µGy min^−1^) in Orion (Fig. 2a). Differences in the inner-belt dose rates validate the shielding design of Orion for large solar-particle events. Orion controls the total dose equivalent from a large solar-particle event to at most 150 mSv (refs. ^22,23^) inside the ‘storm shelter’ in which the HERA HSU1 is located (Extended Data Fig. 4). The HERA HSU2 is located in the crew cabin. The measurement difference in peak dose rates between these two locations was 287 µGy min^−1^ to 134 µGy min^−1^ or a factor of 2, a factor reproduced by simulations (Supplementary Table 1). Large solar-particle events^24,25^ and the inner belt have similar peak fluxes, predicted dose rates, spectral shapes and consist almost entirely of protons (Supplementary Fig. 4). Therefore, our result serves to validate the shielding design for solar-particle events. Simulation of the reference October 1989 solar-particle event shows a fourfold peak dose rate difference between HERA detectors in the crew and the ‘storm shelter’ of 414 µGy min^−1^ to 95 µGy min^−1^, with the discrepancy attributable to the more energetic spectrum of the inner belt (Supplementary Fig. 5 and Supplementary Table 1).

Following the belt transit, Orion spent 25 days in the interplanetary galactic cosmic ray (GCR) environment. Dose rates were similar for all instruments (Fig. 2b,c), demonstrating the small effect that shielding has on GCR absorbed doses, especially compared with the large differences seen in the belt doses. Overall exposure was dominated by GCRs (Fig. 2e), although the inner proton belt (Fig. 2d) contributed up to 23% to the total cumulative dose for EAD MU01 (Extended Data Tables 1 and 2 for cumulative inner and outer belt, GCR and mission doses). No solar-particle events occurred during the Artemis I mission and the only changes in the environment were dose reductions of the GCR values of a third owing to the solid angle blocked by the Moon during close approaches (Fig. 2f,g).

**Fig. 3:**
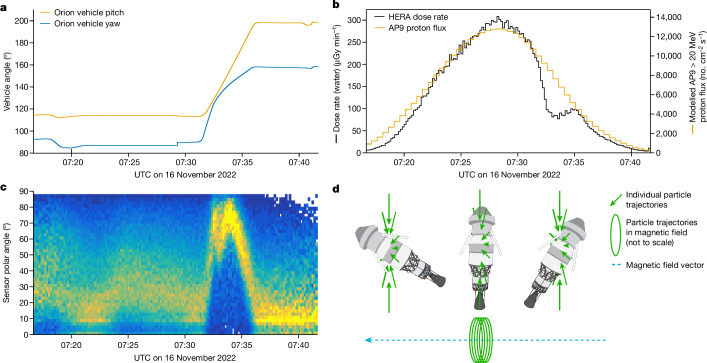
Unexpected dose decrease during the inner-belt pass. **a**, Orion spacecraft pitch (up and down with respect to the nose/docking adapter of the Orion capsule) and yaw (left and right) angle. **b**, Measured absorbed dose rate from HERA HSU2 and modelled AP9-IRENE proton flux. **c**, Measured particle polar angle distribution from HERA HSU2. Ninety degrees corresponds to the long axis of the spacecraft. **d**, The Orion spacecraft with upper stage attached shown in relation to the magnetic-field vector and the particle trajectories. Individual particles rotate in tight spirals around the magnetic-field line, forming a ‘plane’ of radiation. The observed dose-rate drop is interpreted as the vehicle rotating its heavily shielded axis through this plane. Source Data

**Extended Data Fig. 5:**
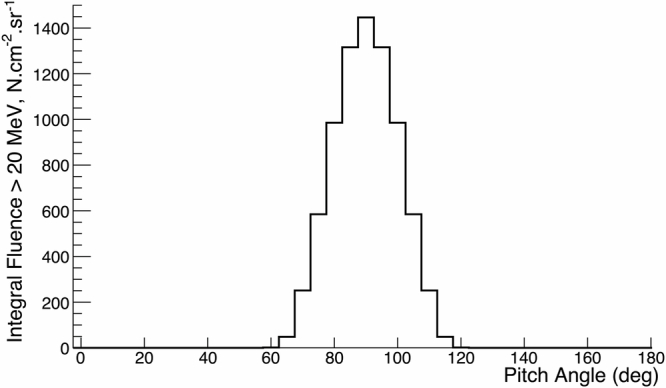
Modelled proton pitch-angle distribution for Artemis I inner-belt transit. The pitch-angle distribution was calculated with the AP9-IRENE radiation belt model run over the Artemis I trajectory through the inner proton belt.

Towards the end of the inner-belt transit, Orion performed a 90° rotation lasting 5 min to execute trans-lunar injection (Fig. 3a and Supplementary Fig. 6). During this manoeuvre, an unexpected decrease in the dose rate of 50% was observed in comparison with AP9-IRENE^26^ calculated proton fluxes (Fig. 3b). Measurements of the proton pitch angle by HERA show a 90° shift towards the long axis of Orion (Fig. 3c). This axis is more shielded owing to the dorsal airlock and ventral Artemis I second stage. AP9 modelling (Extended Data Fig. 5) and HERA measurements (Fig. 3c) show that the inner-belt protons have pitch angles perpendicular to the Earth’s magnetic field and so particle flux comes from a quasi-2D plane perpendicular to the magnetic field. Here we conclude that the dose reduction originated from the rotation of bulkier parts of Orion into the proton plane (Fig. 3d). This shows that preferential spacecraft orientations, in which the thickest shielding is placed into the path of incoming radiation, have the potential for large reductions in radiation exposure in directional fields. These include orbits with frequent belt passes, such as an Artemis abort scenario, as well as the onset of solar-particle events^27^.

Measurements with M-42 (Table 1) inside and outside the MARE Helga phantom show that inner proton belt doses vary roughly twofold from the least (right lung) to the most (spine) shielded internal organs and the front skin dose has a threefold increase compared with the spine. Inner proton belt pass measurements also showed a 20% difference between the left and right lungs, probably because of local shielding and radiation field directionality. Similar to the external measurements, GCR cumulative organ doses vary by only a few percent, for which the back skin with the highest shielding has the highest dose.

**Table 1:** Absorbed dose values inside and outside the MARE Helga phantom

M-42		Cumulative absorbed dose *D* (mGy)		
No.	Location	Inner belt	Outer belt	GCR
SN126	Skin (front)	1.96 ± 0.22	0.11 ± 0.01	9.38 ± 1.03
SN144	Left lung	1.00 ± 0.11	0.09 ± 0.01	9.44 ± 1.04
SN145	Right lung	1.19 ± 0.13	0.08 ± 0.01	9.44 ± 1.04
SN146	Stomach	0.91 ± 0.10	0.08 ± 0.01	9.42 ± 10.4
SN147	Uterus	0.95 ± 0.10	0.09 ± 0.01	10.07 ± 1.11
SN148	Spine	0.61 ± 0.07	0.06 ± 0.01	9.56 ± 1.05
SN127	Skin (back)	0.80 ± 0.09	0.07 ± 0.01	10.17 ± 1.12

Cumulative absorbed dose (*D* in mGy) split by flight phases (Fig. 1c). Flight phases relate to the crossing of the inner and outer radiation belts, as well as the GCR environment in free space. Data are provided for seven M-42 detectors mounted on the skin and in the internal organs of the MARE Helga phantom (Extended Data Fig. 1). Absolute errors quoted after ±.

**Extended Data Fig. 6:**
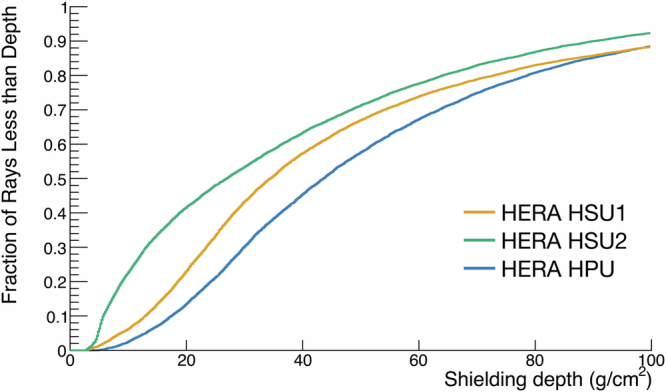
Shielding distributions for the HERA HPU, HSU1 and HSU2 sensors. The shielding distributions were calculated from a detailed computer-aided design model of the Orion vehicle, through which 10,000 equidistant rays were traced from each detector location. The amount of material along each ray was then calculated and normalized to areal density. To obtain the aluminium equivalent thickness, we can divide the *x* axis by the density of aluminium (2.7 g cm^−3^). Owing to its dense construction, Orion is a heavily shielded vehicle, with 80% of rays to the HSU1 location in the storm shelter having more than 20 g cm^−2^ (7.4 cm of aluminium equivalent thickness).

**Extended Data Fig. 7:**
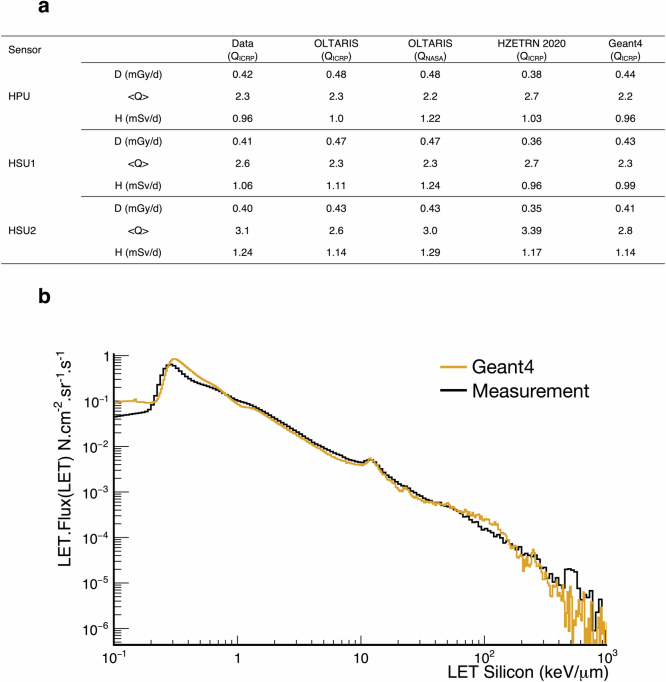
Comparisons of space radiation GCR data and simulations. **a**, Table of relevant dose quantities as the absorbed dose rate (*D* in mGy day^−1^), the mean quality factor <*Q*> and the dose equivalent rate (*H* in mSv day^−1^) as measured with the three HERA sensors and the relevant calculated values applying the OLTARIS, HZETRN and Geant4 codes. Calculations for the HERA sensors are based on the ray-traced shielding locations as provided in Extended Data Fig. 6. **b**, Comparison of Geant4 Monte Carlo-simulated GCR LET spectra in silicon and measured data for the HPU (the most shielded sensor). Note, that LET spectra are shown as a lethargy-style representation to preserve the area-normalization feature of the histogram across the logarithmic *x* axis. Source Data

Modelling tools have not previously been validated in a heavily shielded vehicle in the interplanetary GCR environment and are of great importance for managing radiation on future missions. Detailed models of the local shielding were constructed from CAD models of Orion (Extended Data Fig. 6). We compared Artemis I measurements with four different modelling solutions using the HZETRN^28–31^ and the Geant4 (ref. ^32^) transport codes that transported the Badhwar-O’Neill model^33^ of GCR flux through shielding and show broad agreement with experimental data (Extended Data Fig. 7).

**Fig. 4:**
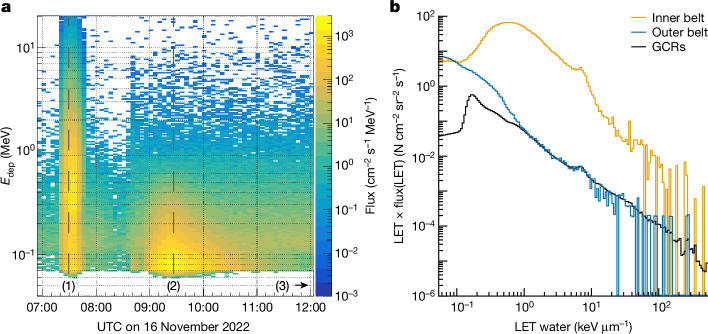
Inner, outer and GCR spectra from M-42 and HERA. **a**, Energy deposition (*E*_dep_, in F cm^−2^ s^−1^ MeV^−1^) spectra measured by M-42 SN126 for the inner (1) and outer (2) belt passes and for the subsequent free-space GCR environment (3). The inner-belt spectra peak at approximately 350 keV energy deposition in Si owing to the dominant proton contribution, whereas the peak for the outer belt is at roughly 70 keV in Si owing to the dominant electron (X-ray) contribution. **b**, LET spectra (in water) for the three flight phases inner belt, outer belt and GCRs, as shown in **a** from the HERA HSU2 instrument. The LET spectra are shown as a lethargy-style representation to preserve the area-normalization feature of the histogram across the logarithmic *x* axis. Source Data

Time-dependent energy-deposition spectra and linear energy transfer (LET) spectra were measured by the M-42 and HERA detectors (Fig. 4a,b). During the outer electron belt transit, M-42 shows maximum peaks in the spectra at energy depositions of about 70 keV (Fig. 4a, (2)). Electrons in the outer belts have extremely short ranges of a few centimetres in water. It is therefore concluded that the radiation environment during the outer-belt pass is mostly composed of secondary Bremsstrahlung (X-rays), as also seen in the HERA data (Fig. 4b) for the outer-belt spectra. Although their dose contribution is small (Table 1 and Extended Data Tables 1 and 2), this is a notable demonstration of the natural variation in space radiation environments.

Calculation of the ICRP60 (ref. ^34^) mean quality factor <*Q*> from the HERA LET spectra (Fig. 4b) provides an estimate of the biological harm of GCRs. The product of <*Q*> and the absorbed dose in Gy yields the biologically relevant dose equivalent in sievert. HERA <*Q*> were 2.30, 2.63 and 3.06, with lower <*Q*> in the more shielded locations (Extended Data Table 1). Calculated dose equivalent rates from GCRs were 0.96–1.24 mSv d^−1^. These results are lower than the data reported by other, more lightly shielded instruments for a similar period of the solar cycle, such as the MSL-RAD (1.58 mSv d^−1^) (Supplementary Fig. 7) or CRaTER (1.55 mSv d^−1^). They are similar to those calculated for interplanetary space from high-latitude measurements inside the International Space Station (1.40 mSv d^−1^) (Table 2). Our data shows that there is a modest decrease in dose equivalent of around 30% for a roughly 80% increase in shielding mass (Extended Data Table 1) (median aluminium equivalent areal density: 25 g cm^−2^ for HSU2 and 45 g cm^−2^ for HPU). This can be compared with the substantial (factor of 2) reduction seen during belt passes. This provides confirmation of modern computational studies showing that, although radiation shielding does not affect the absorbed dose (imparted energy), the biological impact of GCRs can be affected by large amounts of radiation shielding. Total mission dose equivalents were 26.7–35.4 mSv, with between 1.80 and 3.94 mSv attributable to the belt passes (Extended Data Table 1).

**Table 2:** Measured radiation data around the Solar System

Location	*H* (mSv d^−1^)
Johnson Space Center, Houston, USA	0.001
Germany average	0.006 (ref. ^38^)
International Space Station	0.68 (ref. ^39^)
International Space Station (extrapolated free space)	1.40 (ref. ^39^)
Lunar surface	0.81 (ref. ^40^)
Low lunar orbit	1.55 (ref. ^10^)
Mars transit	1.58 (ref. ^41^)
Mars surface	0.65 (ref. ^37^)
Artemis I	0.96–1.24

Average dose equivalent rate (*H* in mSv d^−1^) for the December 2022 time frame for various locations (Earth, the International Space Station and high-latitude extrapolation to free space, Moon surface and Moon orbit, Mars transit and Mars surface), as well as the Artemis I GCR data. The Mars transit data are based on comparable measurements from 2011–2012 during a similar phase of the solar cycle (Supplementary Fig. 7).

The NASA astronaut career dose limit is 600 mSv, known as the ‘NASA effective dose’, a limit protective against cancer at the 3% mean risk of radiation-exposure-induced death for 35-year-old females above the non-exposed baseline mean^23,35^. Overall predicted radiation exposures for upcoming Artemis missions do not approach this limit owing to their similar duration to Artemis I. Artemis I measurements can be extrapolated to exemplary Mars missions^36^ for a similar point in the solar cycle, as the GCR field is approximately constant outside the Earth’s magnetosphere on this scale. The experimentally derived dose equivalents for such a mission are about 30% lower than those reported in the literature so far^37^ and potentially within the 600 mSv limit (Supplementary Materials 5). However, the details of future missions will depend heavily on shielding, trajectory, modulation of GCR with the solar cycle and severity of solar particle events. Orion is a small and massive spacecraft and therefore shows large improvements in radiation exposures compared with measurements on planetary science missions. Radiation protection policy aims to keep exposure ‘as low as reasonably achievable’ (ALARA), which suggests short missions in heavily shielded vehicles. Programmes have good reasons to prefer long missions in light vehicles. It is therefore clear that effective management of radiation risk will remain a key challenge for human space exploration.

## Methods

### DLR M-42 detector

The DLR M-42 battery-powered radiation detector uses a 1.22-cm^2^-area and 300-µm-thick silicon photodiode for the measurements of the space radiation environment. The instrument measures the energy-deposition spectra in the detector applying 210 energy bins (distributed in equidistant logarithmic bins with 75 bins per decade). With this, the energy-deposition range (in Si) for the detector ranges from 0.06 to more than 20 MeV. The highest channel is used as an overflow channel. All energy depositions above the upper threshold are denoted to this channel. For the NASA Artemis I mission, 16 of these M-42 detectors were applied to measure the radiation load either on the surface of the MARE female phantoms (Helga and Zohar) or directly positioned at the most radiosensitive organs (lungs, stomach, uterus, spine). M-42 was powered with two primary batteries for the mission and stored the science and housekeeping (voltage, temperature) data every 300 s in two non-volatile flash memories (primary and secondary). For this study, data from seven of the M-42 instruments were applied, SN126 and SN127 mounted on the surface (skin) and SN144–SN148 positioned in the right and left lungs, stomach, uterus and spine of the radiation phantom Helga. To account for the time after installation in Orion and before the NASA Artemis I launch, the systems were equipped with accelerometer sensors. On installation of the batteries, the systems were in sleep mode. The instruments were woken up as a result of the acceleration sensed following the launch of the NASA Artemis I mission and then began the science data acquisition. M-42 data for the NASA Artemis I mission therefore comprises the energy-deposition spectra (in Si) and the absorbed dose derived from the spectra.

### NASA CAD detector

The NASA CAD battery-powered radiation detector uses Direct Ion Storage (DIS) technology to store the cumulative dose in water (H_2_O). The system setup was done in a way that data are stored either in relevant time intervals (180 to 300 s) and/or when a certain dose threshold was reached during the mission. For the NASA Artemis I mission, a total of 18 of these detectors were applied to measure the dose on the surface (skin) of the MARE female radiation phantoms (Helga and Zohar). For this study, two of the CAD systems were applied, CAD 0082 and CAD 0089, mounted on the surface (skin) of the radiation phantom Helga. Data from the CAD detectors consist of time-resolved absorbed dose rates.

Relevant locations and mounting of the M-42 and CAD instruments on and inside the MARE radiation phantom Helga is given in Extended Data Fig. 1.

### ESA EAD detector

The ESA EAD battery-powered radiation instrument comprises four radiation detectors: two 0.3-cm^2^-area silicon (Si) detectors with thicknesses of 300 µm and 7 µm, one DIS detector and a radiation-sensing field-effect transistor (RadFET). In this study, the focus was on the data from the combination of the thick and thin silicon diode. The relevant energy-deposition ranges (in Si) are 0.055–16.496 MeV for the 300-µm-thick diode and 0.194–27.613 MeV for the 7-µm-thin diode. The energy depositions are stored in 32 equidistant logarithmic bins each for both diodes. For the determination of the absorbed dose in Si, the two diode datasets are combined to account for overlapping energy-deposition regime, thereby applying 26 channels from the thick diode in combination with 31 channels from the thin diode. This also allows us to determine the LET spectra combining the thick and thin diodes for a LET range in H_2_O from 0.09 to 1,470 keV µm^−1^. For the NASA Artemis I mission, five of these units were mounted inside the Orion spacecraft. Each unit was equipped with two primary batteries. Relevant science and housekeeping data were stored every 300 s during the mission. For this study, data from two of the EADs was applied, EAD MU01 mounted on the wall of Orion and EAD MU04 mounted in the storm shelter of Orion. To account for the time after installation in Orion and before launch, the system was equipped (as for the M-42) with an accelerometer sensor. On installation of the batteries, the system was in sleep mode. The instruments were woken up as a result of the acceleration sensed following the launch of the NASA Artemis I mission and then began the science data acquisition. Extended Data Figs. 2 and 3 provide the location of the units inside the Orion capsule.

### NASA HERA detector

The NASA HERA detector is the flight radiation detector for the Orion spacecraft. It consists of three separate sensors (the HERA Processing Unit (HPU) and two HERA Sensor Units (HSU1 and HSU2)). Each HERA sensor contains a Timepix hybrid pixel detector^42,43^. The Timepix detector consists of 256 × 256 pixels of 55-μm pitch for a total area of 1.4 × 1.4 cm (about 2 cm^2^). The salient feature of hybrid pixel detectors is that each individual pixel contains a full electronic pulse processing chain including preamplifier, shaper, threshold discriminator and analogue-to-digital converter fit into the footprint of the overlying semiconductor pixel. The effect of the matrix of pixels is that traversing particles create characteristic ‘tracks’ or ‘clusters’ in the sensor, which can be processed to reveal information about the crossing particle^44^. These quantities include track geometry such as the polar/pitch angle, track length and deposited energy. In turn, the per-particle stopping power d*E*/d*x* can also be calculated. Each Timepix is calibrated over a wide energy range from a minimum detectable threshold of 5-keV X-rays through to heavy ions of 500 keV µm^−1^ to ensure performance measuring the GCR environment^45–47^. For high-dose-rate proton events such as solar-particle events or radiation belts crossings, each sensor is acceptance tested with a proton beam at dose rates between 10 µGy min^−1^ and 10 mGy min^−1^ at the Chicago Proton Center in Naperville, IL, USA. The HERA system is hard-mount integrated into the Orion spacecraft. HERA reports processed engineering, science, display and caution and warning telemetry to the Orion vehicle, which sends it on to mission control. It also saves raw data onto an onboard 8-GB storage drive for post-mission analysis. The complete HERA system weighs roughly 1.5 kg and consumes 8 W of power. HERA was automatically powered up by the Orion flight computers at 50,000-ft altitude and then remained powered until shortly before re-entry, when it was shut off. HERA sent telemetry on a minute-wise cadence to the vehicle and mission control in Houston throughout the mission. HERA also saved higher-resolution raw frame data in its onboard storage for post-mission analysis. HERA data consist of per-minute dose and LET spectra, as well as detailed pixel data and per-‘cluster/track’ data. Extended Data Fig. 4 provides pictures of the HERA instruments.

### Cumulative shielding distributions of HERA instruments in the Orion crew cabin

The distribution of shielding around sensors in Artemis I was determined from the available high-fidelity CAD models of the Artemis I Orion crew capsule (Extended Data Fig. 6). Ten thousand evenly distributed rays were cast at each sensor position and the thickness, density and type of material intersected by each ray is tabulated. Materials are subdivided into aluminium-like and polyethylene-like materials based on density, and each ray is assigned an aluminium and polyethylene areal density (units of g cm^−^^2^). We note the general reliability of these models during the NASA Artemis I spaceflight as the ‘crew’ did not move during the mission and no mass was moved in or out of the storage lockers. This analysis was only carried out for the NASA HERA instruments, which largely bracket the radiation environment in the crew cabin.

### Simulation methodologies

OLTARIS is an online tool available at https://oltaris.nasa.gov (ref. ^31^). It provides a convenient interface for running HZETRN simulation studies of the space radiation environment and its interaction with shielding. OLTARIS simulations described in the main text used a single spherical shell with the detector shielding depth as an approximation for the shielding distribution of Orion. The Badhwar-O’Neill 2020 (BON2020)^33^ GCR flux model was used to create the primary energy spectrum. OLTARIS was set to output dose in tissue and dose equivalent in tissue using both the ICRP60 quality factor and the NASA quality factor. The geometry used was set to ‘Sphere’, allowing for computation with the latest 3DHZETRN^28,29^ transport code. 1DHZETRN^29^ was used to transport the input BON2020 GCR environment through a detailed shielding model of the Orion vehicle. In HZETRN, a two-layer lookup table is generated by transporting particles in 1D through varying thicknesses of aluminium and polyethylene slab layers. The particle flux versus depth table is multiplied by a material stopping power table for silicon to generate a dose versus depth lookup table. The point dose for each sensor is derived by interpolating the lookup table and computing dose for the areal density of each ray, then taking the sum of each ray’s particle flux contribution weighted by its solid angle. The Geant4^32^ simulation used for this article used cosine radiation generated from a 15-cm-diameter spherical shell to generate isotropic space radiation. The input particle spectra were generated from the BON2020 model ran over the Artemis I mission, and these were generated in Geant4 by using the Geant4 General Particle Source. These spectra were transported through an 11-cm-diameter sphere aluminium shell, in which the density was varied over different runs to account for differing thicknesses of shielding around the HERA instrument. The resultant LET and particle spectra were assumed to be a linear combination of the above runs (that is, for a hypothetical shielding distribution with 10% 5 g cm^−^^2^ and 90% 10 g cm^−^^2^, the results were weighted by 90% of the 10-g simulation and 10% of the 5-g simulation). The Livermore model was used to model electromagnetic physics and the INCLXX model for hadronic physics. The physics cut was set at 2 μm. The Timepix sensor was modelled as a simple silicon slab and the resultant energy depositions in the sensor digitized. To simulate the per-particle tracking style measurements of the Timepix, the track length was calculated from the individual energy-deposition events in the Monte Carlo simulation and the d*E*/d*x* was scored on a per-particle basis. To ensure a reasonable number of hits in the Timepix from low LET particles, a step limit of 5 μm was enforced. The Timepix sensor has dimensions 14 mm × 14 mm × 500 μm, so these limits were considered much smaller than the geometry and therefore reasonable. Doses in water and quality factors were calculated as per the main text (that is, with a flat 1.24 conversion factor). The Geant4 code is available from the authors on request, along with macros for input particle spectra and combination of simulation results. Results for the simulations are provided in comparison with measured data in Extended Data Fig. 7.

### Estimation of uncertainties in dose rate and ICRP60 quality factors

We estimate that the systematic error in the dose measurement is 10% for all detectors. Previous comparisons of various radiation detectors onboard the International Space Station generally show agreement within 5–10%. For the HERA detector in particular during instrument acceptance testing at a proton facility, we levy a requirement that our flight instruments agree with dose measurements from an independently calibrated ion chamber calibrated to dose in water over the energy range 100–200 MeV with a National Institute of Standards and Technology (NIST) traceable Cs-137 source to within 10%. 10% is a conservative estimate and this uncertainty captures much of the variation in different instrument-calibration techniques. However, the authors note that, in detailed comparisons, the silicon detectors (M-42, EAD and Timepix) generally agree with each other at a much higher precision than 10%.

The silicon-to-water (Si/H_2_O) conversion factor is a subject of some discussion for space-radiation measurements. We use a factor of 1.24 for these results, the same as that used by us previously for the REM detectors onboard the space station and the BIRD detector, and similar to the DLR/CAU DOSTEL instruments. Following ref. ^8^, we set the uncertainty on this conversion to 5%. The dose rate systematic error is found by combining the 10% and 5% errors in quadrature and is estimated as 11.1%. The statistical error in the dose-rate measurement for all detectors over the whole mission is extremely small.

The error in the quality factor is found by means of Monte Carlo bootstrapping. Here the full list of hits was resampled on the basis of an attempt to realistically quantify the errors in our measurement. Three kinds of errors were assumed. The first was those corresponding to the systematic errors, which were applied with the same weight as a single run of the Monte Carlo. The second is counting (Poissonian) errors in the histogram bin counts and the third is those that are applied on a per-hit/particle basis, namely, the energy resolution of the hit that is assumed to be 10% for low LET particles and 30% for particles with LET > 50 keV μm^−1^, in line with the loss of energy resolution from the high LET calibration of the Timepix detectors^47^. The fractional error in the dose equivalent rate was found by combining the dose rate and quality factor fractional errors in quadrature. The above information is summarized in Extended Data Table 3.

## Online content

Any methods, additional references, Nature Portfolio reporting summaries, source data, extended data, supplementary information, acknowledgements, peer review information; details of author contributions and competing interests; and statements of data and code availability are available at 10.1038/s41586-024-07927-7.

## Supplementary information

Supplementary Information Supplementary Materials 1–5 Figs. 1–7, Tables 1 and 2, and References 1–24.

## Source data

Source Data Fig. 2

Source Data Fig. 3

Source Data Fig. 4

Source Data Extended Data Fig. 7

## Figures and Tables

**Extended Data Table 1:**
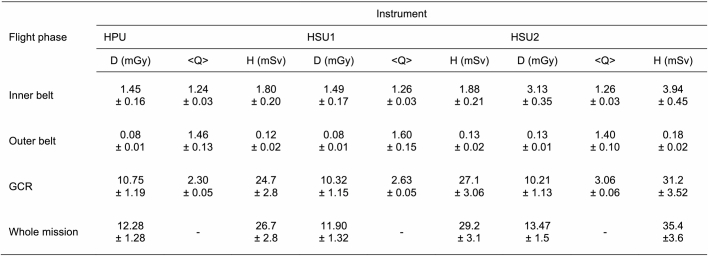
HERA dose quantities split by phase of flight

The flight phases relate to the crossing of the inner and outer radiation belts, as well as the GCR environment in free space and the whole mission. Provided is the relevant cumulative absorbed dose (*D*), the mean quality factors <*Q*> and the cumulative dose equivalents (*H*) split by phase of flight. Relevant mounting locations are provided in Extended Data Fig. 4. Absolute errors quoted after ±.

**Extended Data Table 2:**
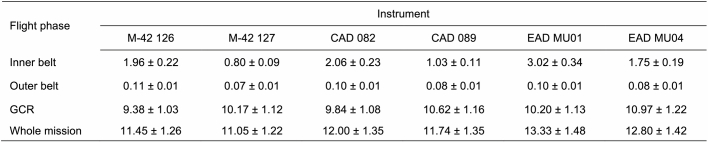
M-42, CAD and EAD dose quantity split by phase of flight

The flight phases relate to the crossing of the inner and outer radiation belts, as well as the GCR environment in free space and the whole mission. Provided is the relevant cumulative absorbed dose (*D* in mGy) split by phase of flight. M-42 SN126 and CAD 0082 are positioned on the skin (front) and M-42 SN127 and CAD 0082 are mounted on the skin (back) of the Helga phantom (Extended Data Fig. 1). EAD MU01 is located on the wall of Orion, whereas EAD MU04 is mounted in the storm shelter (Extended Data Figs. 2 and 3). Absolute errors quoted after ±.

**Extended Data Table 3:**
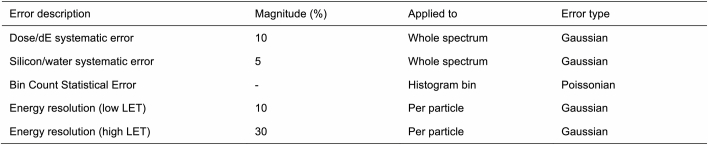
Sources and magnitudes of errors for quality-factor calculation

The quality factors for absorbed dose to dose equivalent conversion reported in this article are calculated using the ICRP60 LET based formalism and the reported LET spectrum/histogram measured by the HERA instrument. This analysis attempts to realistically model all error contributions to the quality factor calculation. The quoted systematic dose error of 10% is based on instrument intercomparisons and is generally considered conservative as the HERA and M-42 instruments tend to agree much better than this. The silicon water conversion error is based on prior Monte Carlo simulation work in the literature (see Methods). The bin count error is based on Poissonian statistics and energy resolution on experimental measurements of the HERA system. The errors shown above are applied to the calculation of ICRP60 quality factor using Monte Carlo bootstrapping/resampling and the reported error in quality factor is the standard deviation of the resultant distribution of calculated quality factors.

## Data Availability

All datasets generated and/or analysed during this study are available from the corresponding authors on reasonable request. The authors intend to make all data publicly available, pending concurrence from the Orion programme, on the NASA Open Science Data Repository RadLab at https://visualization.osdr.nasa.gov/radlab/gui/overview/. Source data are provided with this paper.

## Code availability

All codes applied for analysis during this study are available from the corresponding authors on reasonable request.
