# Supplementary material for: Space radiation measurements during the Artemis I lunar mission
Source: Nature. 2024 Sep 18;634(8032):48–52. doi: 10.1038/s41586-024-07927-7 (PMC11446838; doi:10.1038/s41586-024-07927-7)
Supplement: Supplementary file 1 — Supplementary Materials 1–5 Figs. 1–7, Tables 1 and 2, and References 1–24. [file 41586_2024_7927_MOESM1_ESM.pdf]

nature portfolio

https://doi.org/10.1038/s41586-024-07927-7

Supplementary information

Space radiation measurements during the Artemis I lunar mission

In the format provided by the authors and unedited

Space radiation measurements during the Artemis I lunar mission

Stuart P. George[1,2*], Ramona Gaza[1,3], Daniel Matthiä[4], Diego Laramore[1,3], Jussi Lehti[5], Thomas Campbell-Ricketts[1,3], Martin Kroupa[1,3†], Nicholas Stoffle[1,3⇑], Karel Marsalek[4], Bartos Przybyla[4], Mena Abdelmelek[1,6], Joachim Aeckerlein[4], Amir A. Bahadori[1,2‡], Janet Barzilla[1,3], Matthias Dieckmann[7], Michael Ecord[1,3], Ricky Egeland[1,2], Timo Eronen[5], Dan Fry[1,2], Bailey H. Jones[14], Christine E. Hellweg[4], Jordan Houri[8], Robert Hirsh[3], Mika Hirvonen[5], Scott Hovland[7], Hesham Hussein[9], A. Steve Johnson[1,3], Moritz Kasemann[4], Kerry Lee[1,2¶], Martin Leitgab[1,3&], Catherine McLeod[1,2], Oren Milstein[10], Lawrence Pinsky[11], Phillip Quinn[1,3], Esa Riihonen[5], Markus Rohde[4], Sergiy Rozhdestvenskyy[1,3], Jouni Saari[5], Aaron Schram[12], Ulrich Straube[13], Daniel Turecek[1], Pasi Virtanen[5], Gideon Waterman[10#], Scott Wheeler[2], Kathryn Whitman[1,6], Michael Wirtz[4], Madelyn Vandewalle[2], Cary Zeitlin[1,3], Edward Semones[1,2], Thomas Berger[4*]

AFFILIATIONS

[1]Space Radiation Analysis Group, Johnson Space Center, Houston, TX, USA

[2]National Aeronautics and Space Administration (NASA), Houston, TX, USA

[3]Leidos, Space Exploration and Mission Operations, Houston, TX, USA

[4]German Aerospace Center (DLR), Institute of Aerospace Medicine, Radiation Biology Department, Cologne, Germany

[5]Aboa Space Research Oy (ASRO), Turku, Finland

[6]KBR, 2400 E. NASA Pkwy, Houston, Texas, 77058, USA

[7]European Space Agency (ESA), European Space Research and Technology Centre (ESTEC), Noordwijk, the Netherlands

[8]StemRad Inc., Tampa, FL, USA

[9]Lockheed Martin Space, Houston, USA

[10]StemRad Ltd., Tel Aviv, Israel

[11]University of Houston Department of Physics and Astronomy, 4800 Calhoun Rd, Houston, TX, 77204, USA

[12]CACI, 2100 Space Park Dr, Suite 210, Houston, TX 77058, USA

[13]European Space Agency (ESA), European Astronaut Center (EAC), Cologne, Germany

[14]Oceaneering Space Systems, Houston, TX, USA

[†]Now at: Los Alamos National Laboratory, ISR-1, Bikini Atoll Rd, Los Alamos, NM, United States, 87545, USA

[‡]Now at: Alan Levin Department of Mechanical and Nuclear Engineering, Kansas State University, Manhattan, KS 66506, USA

[¶]Now at: The Aerospace Corporation, Houston, TX, USA

[&]Now at: Abbott Laboratories, Dallas, TX, USA

[#]Now at: Advanced Medical Physics, Inc. Houston, TX, USA

[⇑]Now at: Axiom Space, Houston, TX, USA

*Corresponding authors: stuart.george@nasa.gov ; thomas.berger@dlr.de

Supplementary Materials 1: Overview of Timepix and dose and linear energy transfer (LET) measurement with Timepix

The Timepix[1] hybrid pixel detectors used in HERA[2] consist of a pixelated 500µm thick silicon sensor bump bonded to an underlying Timepix ASIC. The sensor consists of 256 x 256 pixels of 55µm pitch for a total area of 1.4 x 1.4 cm (~2cm^2). The salient feature of hybrid pixel detectors is that each individual pixel contains a full electronic pulse processing chain including preamplifier, shaper, threshold discriminator and analogue to digital converter fit into the footprint of the overlying semiconductor pixel as shown in Supplementary Fig. 1.

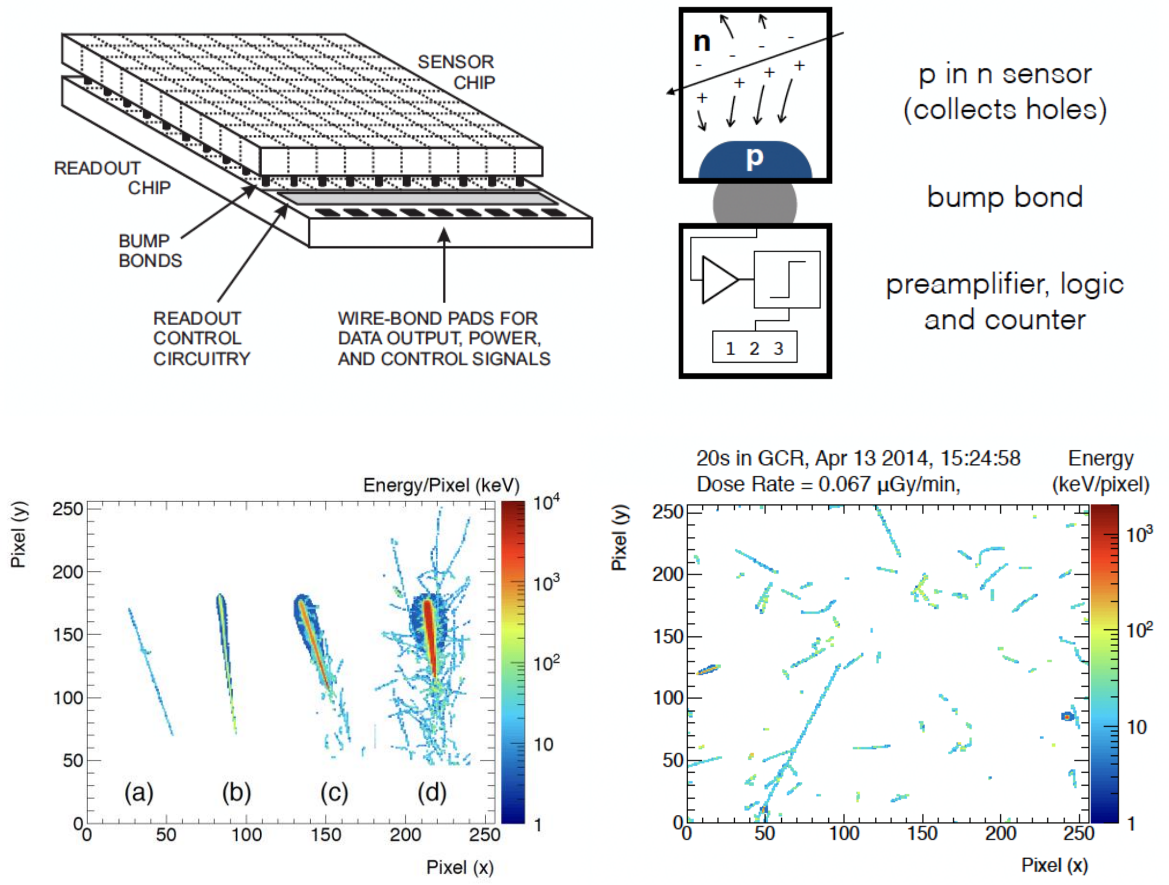

Supplementary Fig. 1 | Hybrid pixel detector geometry (top), example measurements of different traversing tracks from protons to Iron with increasing stopping powers (bottom left), example measurement from International Space Station hybrid pixel detector in 2014 (bottom right).

In the case of the Timepix sensor the pixel array is calibrated to per particle energy deposits with x-ray photons 5.9 – 60 keV, protons of 5 MeV and heavy ions to ensure a uniform energy measurement response across the galactic cosmic ray linear energy transfer (LET) spectrum[3,4]. For higher LET particles, the Timepix suffers from the so called “Volcano” effect, a saturation in the pixel front end which can be reliably calibrated on a per track basis[5].

The effect of the matrix of pixels is that traversing particles create characteristic ‘tracks’ or ‘clusters’ in the sensor which can be processed to reveal information about the crossing particle (Supplementary Fig. 1)[6]. Of particular interest is the per particle chord or track length which can be used to calculate the stopping power dE/dX on a particle by particle basis[7–10].

The calculated absorbed dose is then simple the ratio of the measured energy deposit in the sensor divided by the sensor mass. Each Timepix sensor intended for flight is dose rate tested in a proton beam at Chicago Proton Center in Naperville, Il at a variety of different dose rates against an ion chamber calibrated with a NIST traceable Cs-137 source as well as with a variety of heavy ions at the NASA Space Radiation Laboratory at Brookhaven National Lab, NY. An example of a simulated ‘belt’ pass measurement showing dose rate consistency across a wide range is shown in Supplementary Fig. 2.

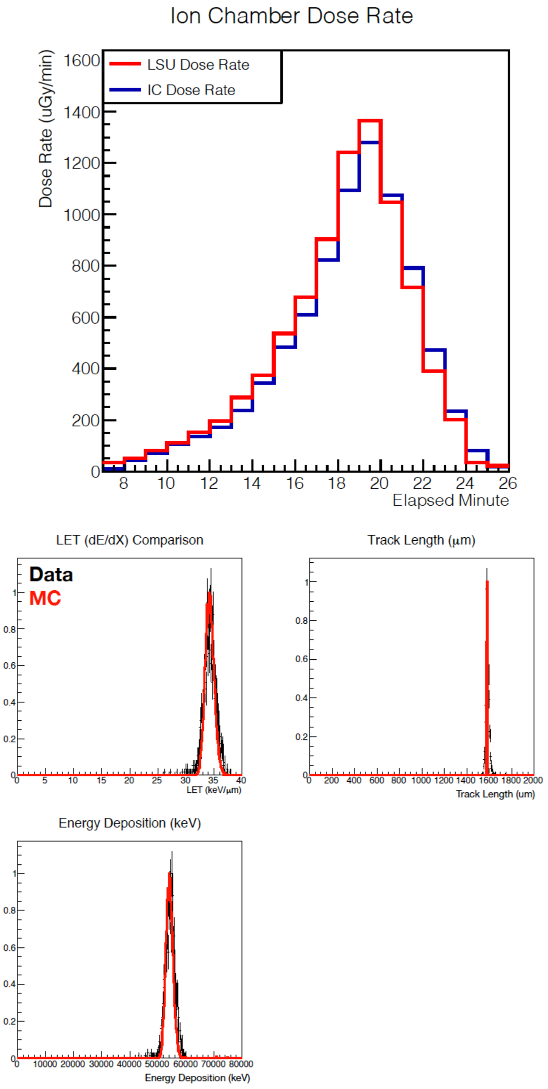

Supplementary Fig. 2 | Example measurements with Timepix hybrid pixel detector (top panel) Timepix “LSU” sensor per minute dose rates compared with externally calibrated ion chamber measured in a DC proton beam at Chicago Proton Center in Naperville, Il. (bottom panels) Example of LET measurement (black) of Oxygen 430 MeV/A ion LET (dE/dX) broken out into measurement of chord length (dX) and energy deposition (dE) compared with Monte Carlo simulation (red).

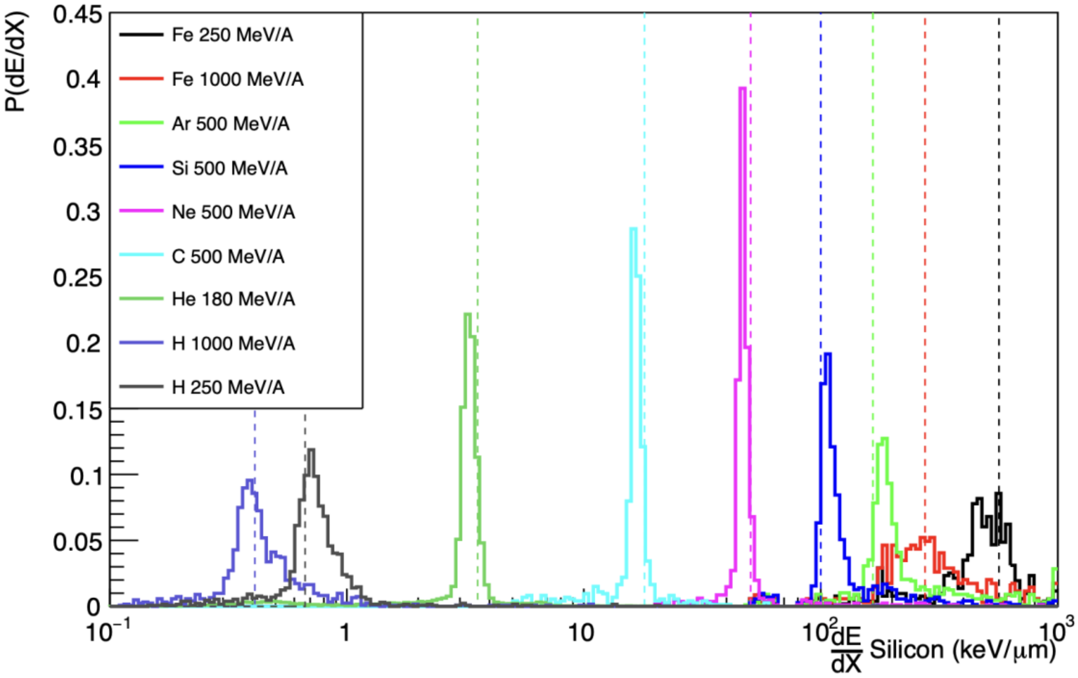

Supplementary Fig. 3 | Example LET measurements with calibrated Timepix detector for a variety of ion species compared to theoretical most probable value (dotted lines).

With appropriately tuned data processing algorithms the pixel detectors can measure dE/dX roughly with the same accuracy as the Landau Vavilov distribution[11], for example see Supplementary Fig.2 which compares experimental beamline data for an oxygen beam with a Geant4 Monte Carlo simulation of energy deposition in a 'perfect' silicon sensor (i.e. one without digitization effects). Example dE/dX measurements are shown in Supplementary Figs. 2-3.

Supplementary Material 2: Comparison of 1989 and Orion VAB Proton Spectra

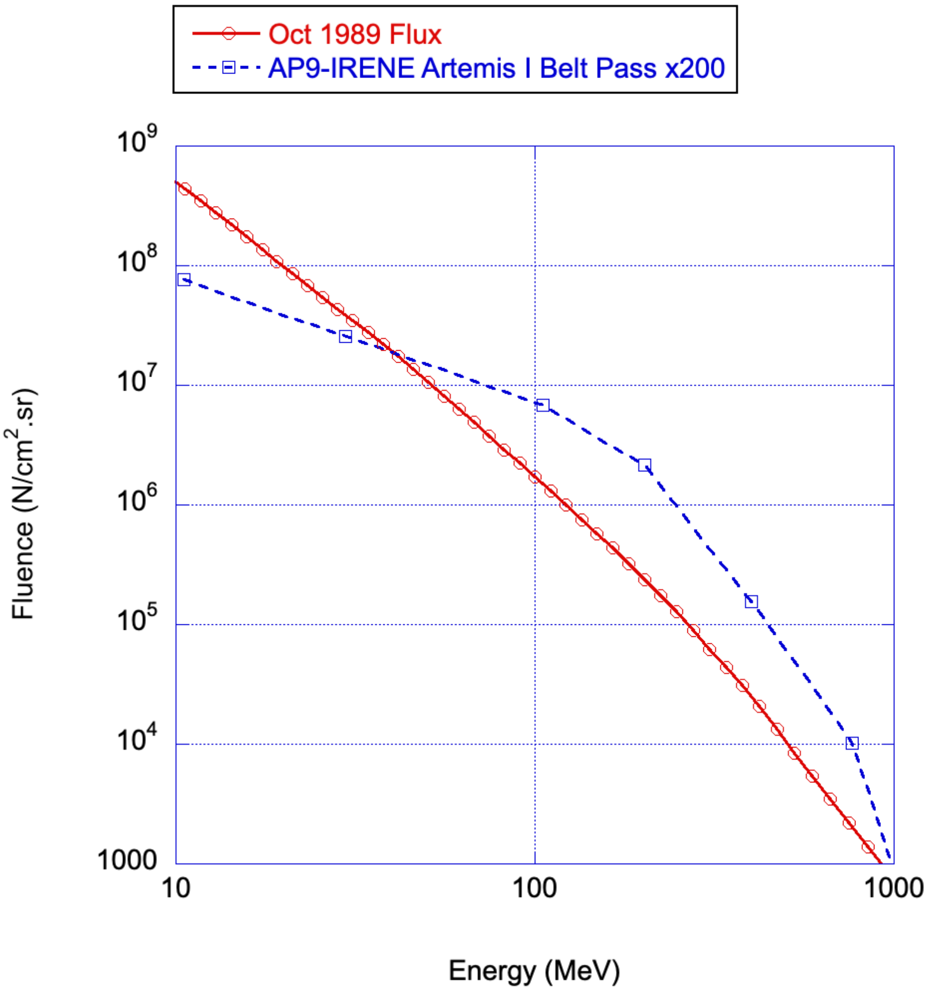

Supplementary Fig. 4 | Comparison of October 1989[12] solar particle event integrated fluence spectrum vs AP-9 model[13] of Artemis I event integrated belt pass scaled by a factor of 200x for comparison purposes.

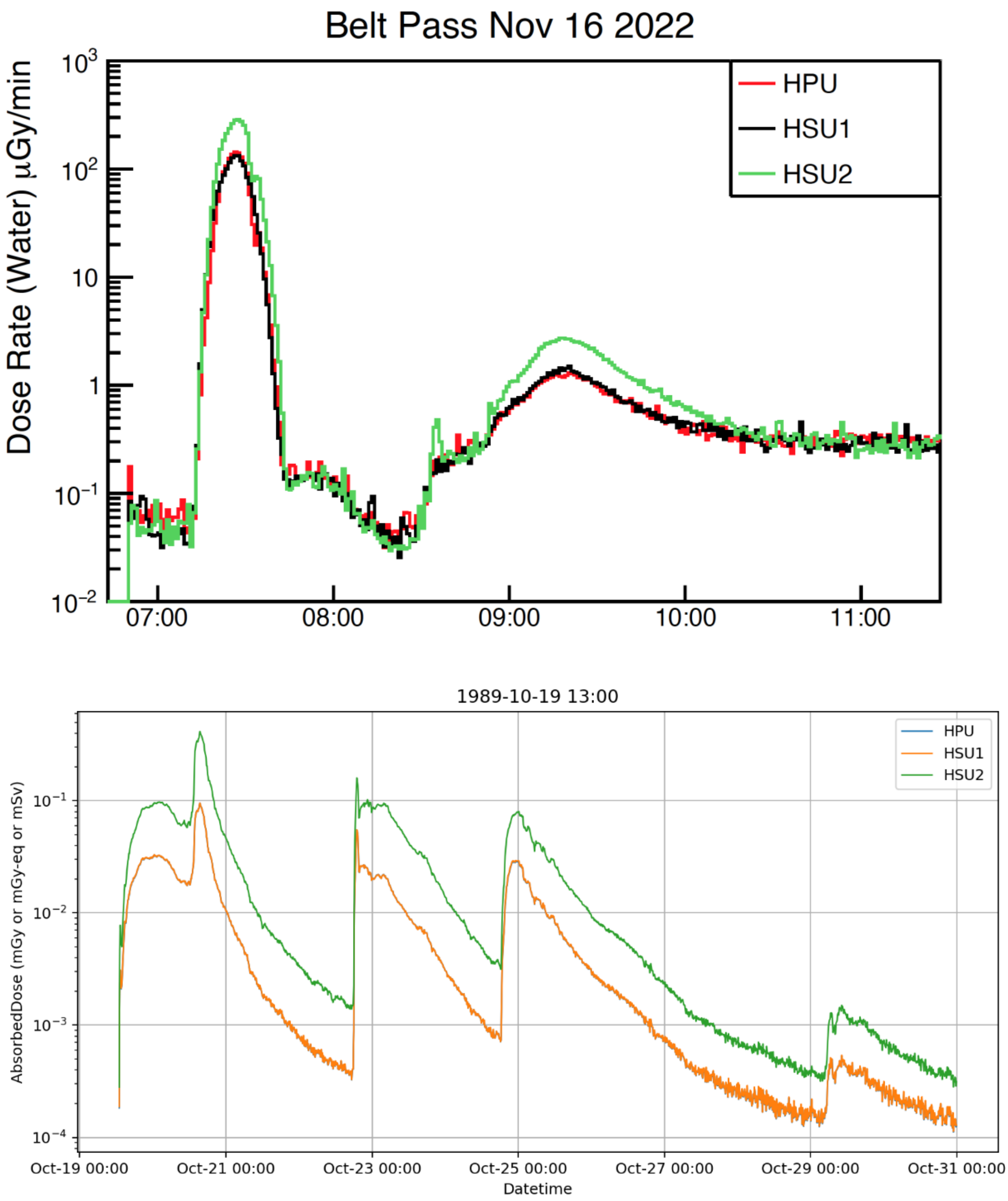

Supplementary Fig. 5 | HERA Artemis I inner belt measurements (upper panel) and simulated (lower panel) October 1989 solar particle event dose rates for HERA locations in Orion shielding. It can clearly be seen that the peak dose rates for the belt pass (287.5 µGy/min) as measured with the HERA HSU2 unit (upper panel) and the October 1989 solar particle event data (414.6 µGy/min) (lower panel) are similar. Note the exceptional length of the October 1989 event (almost 2 weeks) which is the main contributor to the large cumulative doses from this event. Note also, that in this simulation the HPU sensor and HSU1 sensor have very nearly the same dose rates.

Supplementary Table 1 | Model and data comparisons of inner proton belt pass and modelling of large reference SPE

Artemis I Belt Pass	HPU (µGy/min)	HSU1 (µGy/min)	HSU2 (µGy/min)
Measured inner belt pass	144.4	133.54	287.5
Simulated inner belt pass	101.4	104.2	228.9
% of max (HSU2) measured	50.2%	46.4%	100%
% of max (HSU2) simulated	45.5%	44.3%	100%
**Large Reference SPE**	**HPU**	**HSU1**	**HSU2**
Simulated Oct 89 SPE	95.4	95.3	414.6
% of max HSU2 Oct 89 SPE simulated	23.0%	23.0%	100%

Peak dose rates (in water) and modelling comparison for the Artemis I inner belt pass and the Oct 89 solar particle event are provided in Supplementary Table 1.

During the Artemis I inner belt pass the HPU and HSU1 measured peak doses of 50.2% and 46.6% of the maximum as measured with HSU2. This corresponds well with the predicted (simulated) values of 45.5% and 44.3% respectively. Note that the absolute values of the simulated inner belt peak doses vary significantly from the measurement. This is because the radiation belts exhibit considerable variations in particle population levels (the particle energy spectra and pitch angles are determined by the magnetic field) which are not captured in the AP9-IRENE model.

Fractional peak dose reductions using the same shielding model show that the HPU in a similar shielding configuration as the Orion storm shelter would exhibit dose rates 23% of the HSU2 in a comparatively lightly shielded location on the vehicle wall for the Oct 89 solar particle event. Note that peak dose reduction is used as it is unlikely that crew would shelter for the entirety of a large SEP event due to the time duration (e.g. ~12 days for October 1989). Instead they would shelter during the most intense phases which are much briefer in duration.

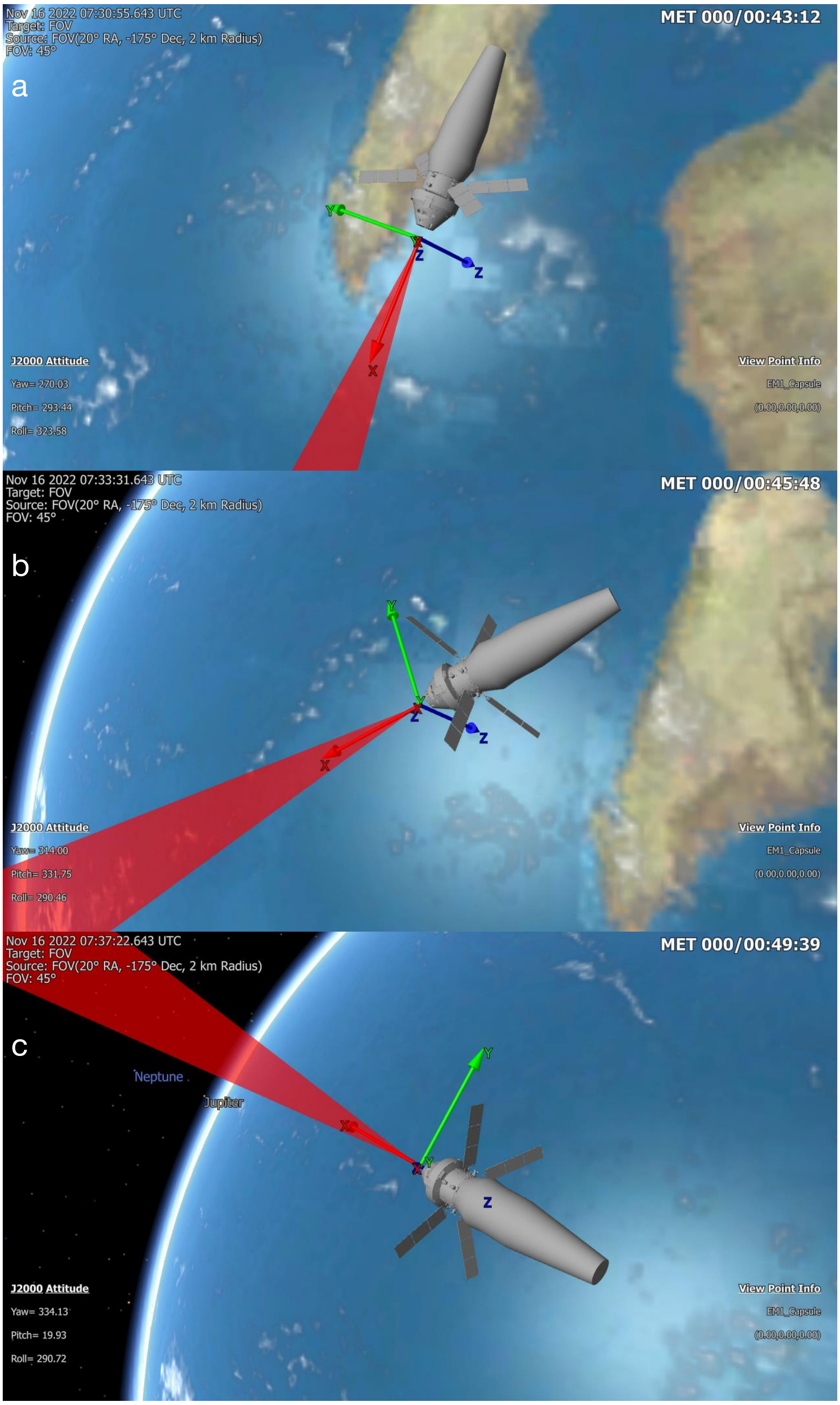

Supplementary Fig. 6 | Visualization showing vehicle orientation during translunar injection burn reorientation maneuver at a, MET, b, 07:33 and c, 07:37. Note that the Earth is roughly oriented south/north in these images and the visible landmass is the north tip of Madagascar.

Supplementary Materials 3: Solar cycle comparisons MSL RAD vs Artemis I and time of Artemis I in solar cycle

The MSL cruise and Artemis I occurred during similar solar conditions. The start date of both missions was almost exactly 1 solar cycle (11 years) apart (Dec 2011 vs Nov 2022) and similar amounts of time after the start of the solar cycle (3 years). Both ACE CRIS[14] Oxygen flux and Oulu neutron[15] counts (Supplementary Fig. 7) are very similar for the measurement period. The overall calculated Phi parameter using the Badhwar O'Neill 2020 code for the Artemis I mission was 557 and for MSL Cruise was 606. It can be seen from the ACE CRIS flux that this is roughly in the midpoint of the solar cycle, neither in solar minimum or maximum.

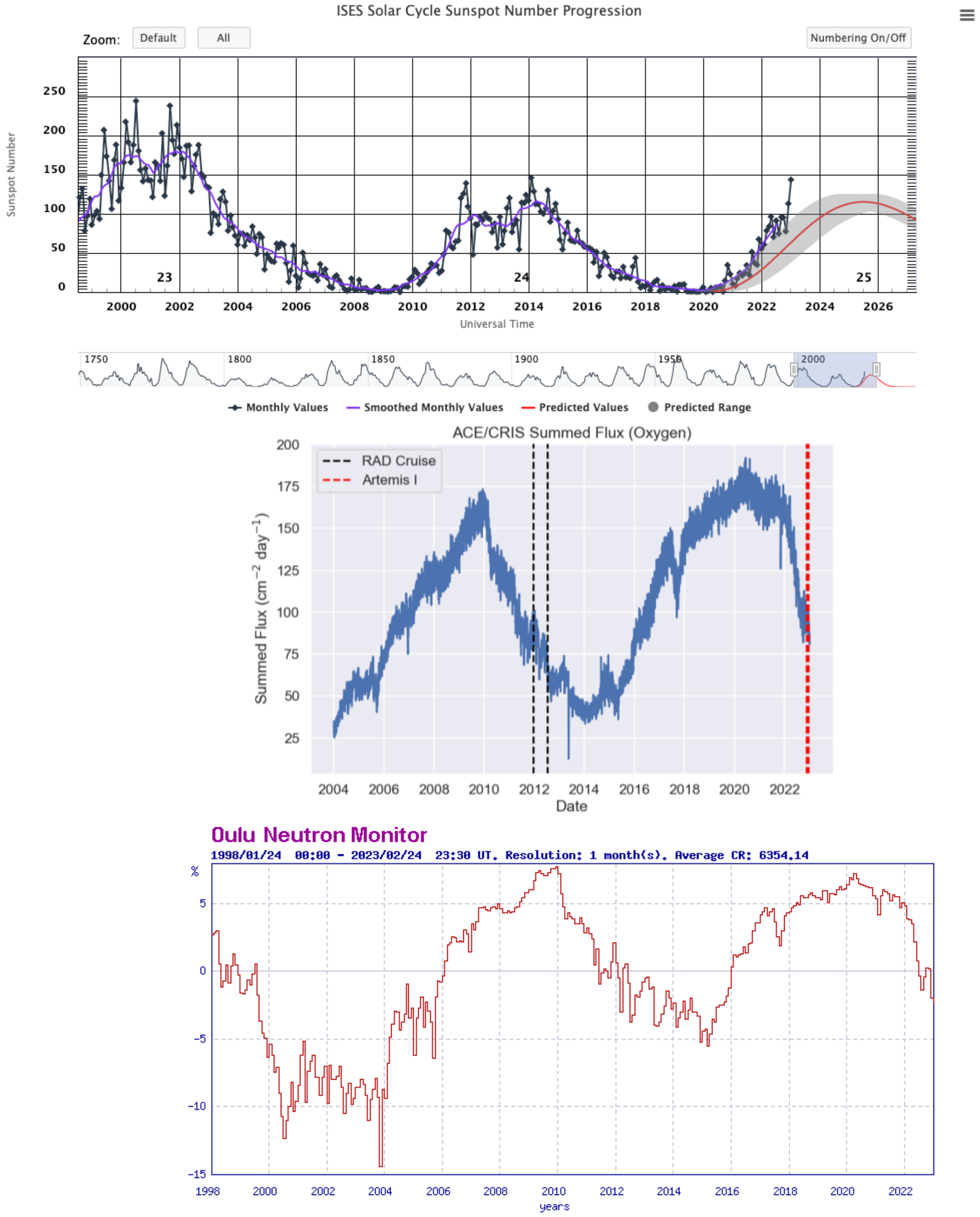

Supplementary Fig. 7 | Progression of solar cycle 23, 24 and 25 in terms of sunspot numbers (top), ACE CRIS Oxygen flux (middle) and Oulu neutron counts (bottom).

Supplementary Materials 4: Notes on NASA Effective Dose and ICRP 60 Dose Equivalent

To formally assess radiation exposure for astronauts, NASA uses the NASA effective dose[16–18]. This differs from the measured dose equivalent in that it is a computationally calculated quantity weighted to account for differing doses to organs due to radiation transport, organ radiation sensitivities, age, and sex. NASA effective dose provides in principle a more accurate measure of an individual's risk than the dose equivalent. In some situations, such as solar particle events where organ doses can vary substantially due to self-shielding from the body the NASA effective dose can be quite different to the dose equivalent. However previous simulation studies have indicated that galactic cosmic ray organ doses do not vary substantially in thickly shielded vehicles[19], making dose equivalent measurements such as those performed by HERA during Artemis I very similar to the calculated NASA effective dose.

Supplementary Materials 5: Mars Mission Doses with an Orion Like Vehicle, Calculations and Assumptions

Earlier measurements of GCR by the RAD instrument on the Mars Science Laboratory (MSL) Curiosity rover have estimated a free space dose equivalent rate of 1.6 mSv/day[20,21]. The dose equivalent rates reported inside the Orion capsule are between 0.96 and 1.24 mSv/day. These measurements were taken with a similar solar modulation parameter (BON 2020 ϕ = 557 MV, Artemis I vs ϕ = 606 MV RAD cruise, see Supplementary Fig. 7) and so are directly comparable.
As explained in the main text these differences can be attributed to the much heavier shielding of Orion. Earlier measurements of GCR by the RAD instrument on the Mars Science Laboratory (MSL) on the Martian surface the RAD measured 0.64 mSv/day[22]. The discrepancy can explained by the shielding from the planetary body (blocking half the sky) and the Martian atmosphere.

The Mars Design Reference Architecture has studied many different mission scenarios which can be used to model crew exposures during Mars missions[23,24]. These scenarios generally fall into three categories following on vehicle design and orbital mechanics (transfer window) considerations, examples of which are shown below:

1. Long surface stay (500 days), moderate transit (360 days)
2. Short surface stay (30 days), (fuel efficient) long transit via Venus slingshot (530 days)
3. Long surface stay (700 days), short transit with nuclear propulsion (200 days)

The radiation budget for these scenarios is shown in Supplementary Table 2 assuming measured HERA free space dose rates and MSL RAD dose rates on the surface. In practice this means the assumption of a heavily shielded transit vehicle and a lightly shielded surface habitat.

Supplementary Table 2 | Estimated Mars mission exposures based on free space HERA data. HPU and HSU1 are most representative of Orion crew module.

Scenario	HERA HPU	HERA HSU1	HERA HSU2	Mission Duration
1. Long stay, moderate transit	0.66 Sv	0.69 Sv	0.77 Sv	860 days
2. Short stay, long transit	0.53 Sv	0.58 Sv	0.68 Sv	560 days
3. Long stay, short transit	0.64 Sv	0.66 Sv	0.69 Sv	900 days

Based on our measurements, a trip to Mars may be possible within the current NASA career dose limit of 600 mSv in a spacecraft with similar (i.e. substantial) shielding to the Orion crew module. Surprisingly, employing nuclear propulsion to reduce transit times does not significantly reduce the overall crew dose due to the increased stay times on the Martian surface. The lowest radiation exposure scenario is the one that minimizes the total trip time (short stay, long transit), although the reduction in radiation exposure versus the other scenarios is modest. These scenarios also do not include Solar Particle Event (SPE) exposure and exemplar large SPE's could deliver 200-300

extra mSv in a vehicle with Orion like shielding. These events are rare with 2 of this class (August 1972 and October 1989) having occurred in the space age. It should also be noted that the Venus slingshot scenario brings the vehicle closer to the sun. Under the assumption that Solar Particle event intensity falls off as r^2 any SPE encountered by the crew at Venus solar distance (0.72 AU) would be twice as intense as at the equivalent Earth distance.

Supplementary References

1. Llopart, X., Ballabriga, R., Campbell, M., Tlustos, L. & Wong, W. Timepix, a 65k programmable pixel readout chip for arrival time, energy and/or photon counting measurements. *Nuclear Instruments and Methods in Physics Research Section A: Accelerators, Spectrometers, Detectors and Associated Equipment* **581**, 485–494 (2007).
2. Stoffle, N. N. *et al.* HERA: A Timepix-based radiation detection system for Exploration-class space missions. *Life Sciences in Space Research* (2023) doi:10.1016/j.lssr.2023.03.004.
3. Jakubek, J. Precise energy calibration of pixel detector working in time-over-threshold mode. *Nuclear Instruments and Methods in Physics Research Section A: Accelerators, Spectrometers, Detectors and Associated Equipment* **633**, S262–S266 (2011).
4. Kroupa, M., Campbell-Ricketts, T., Bahadori, A. & Empl, A. Techniques for precise energy calibration of particle pixel detectors. *Review of Scientific Instruments* **88**, 033301 (2017).
5. George, S. P. *et al.* Very high energy calibration of silicon Timepix detectors. *J. Inst.* **13**, P11014 (2018).
6. Holy, T. *et al.* Pattern recognition of tracks induced by individual quanta of ionizing radiation in Medipix2 silicon detector. *Nuclear Instruments and Methods in Physics Research Section A: Accelerators, Spectrometers, Detectors and Associated Equipment* **591**, 287–290 (2008).
7. Hoang, S. *et al.* Data Analysis of Tracks of Heavy Ion Particles in Timepix Detector. *J. Phys.: Conf. Ser.* **523**, 012026 (2014).
8. Kroupa, M. *et al.* A semiconductor radiation imaging pixel detector for space radiation dosimetry. *Life Sciences in Space Research* **6**, 69–78 (2015).
9. Stoffle, N. *et al.* Timepix-based radiation environment monitor measurements aboard the International Space Station. *Nuclear Instruments and Methods in Physics Research Section A: Accelerators, Spectrometers, Detectors and Associated Equipment* **782**, 143–148 (2015).
10. George, S. Dosimetric applications of hybrid pixel detectors. *University of Wollongong Thesis Collection 1954-2016* (2015).
11. Kroupa, M., Bahadori, A. A., Campbell-Ricketts, T., George, S. P. & Zeitlin, C. Kinetic energy reconstruction with a single layer particle telescope. *Appl. Phys. Lett.* **112**, 134103 (2018).
12. Hu, S. & Semones, E. A Multi-source Calibrated GOES Dataset and Solar Radiation Environment Model Update. (2022).

13. O'Brien, T. P. *et al.* Changes in AE9/AP9-IRENE Version 1.5. *IEEE Transactions on Nuclear Science* **65**, 462–466 (2018).

14. Stone, E. C. *et al.* The Cosmic-Ray Isotope Spectrometer for the Advanced Composition Explorer. *Space Science Reviews* **86**, 285–356 (1998).

15. Usoskin, G., Mursula, K., Kangas, J. & Gvozdevsky, B. On-line database of cosmic ray intensities. in *Proceedings of ICRC 2001* vols 1–4 (2021).

16. Cucinotta, F. A., Kim, M.-H. Y. & Chappell, L. J. Space Radiation Cancer Risk Projections and Uncertainties - 2010. (2011).

17. Cucinotta, F. A. Biophysics of NASA radiation quality factors | Radiation Protection Dosimetry | Oxford Academic. *Radiation Protection Dosimetry* **166**, 282–289 (2015).

18. *Space Radiation and Astronaut Health: Managing and Communicating Cancer Risks*. (National Academies Press, 2021). doi:10.17226/26155.

19. Cucinotta, F. A. *et al.* Space Radiation Cancer Risks and Uncertainties for Mars Missions. *Radiation Research* **156**, 682–688 (2001).

20. Zeitlin, C. *et al.* Measurements of Energetic Particle Radiation in Transit to Mars on the Mars Science Laboratory. *Science* **340**, 1080–1084 (2013).

21. Zeitlin, C. *et al.* Measurements of radiation quality factor on Mars with the Mars Science Laboratory Radiation Assessment Detector. *Life Sciences in Space Research* **22**, 89–97 (2019).

22. Hassler, D. M. *et al.* Mars' Surface Radiation Environment Measured with the Mars Science Laboratory's Curiosity Rover. *Science* **343**, 1244797 (2014).

23. Drake, B. G. & Watts, K. D. *Human Exploration of Mars Design Reference Architecture 5.0, Addendum #2 - NASA Technical Reports Server (NTRS)*. https://ntrs.nasa.gov/citations/20160003093.

24. Drake, B. G., Hoffman, S. J. & Beaty, D. W. Human exploration of Mars, Design Reference Architecture 5.0. in *2010 IEEE Aerospace Conference* 1–24 (2010). doi:10.1109/AERO.2010.5446736.
